# Spirituality and health in the curricula of medical schools in Brazil

**DOI:** 10.1186/1472-6920-12-78

**Published:** 2012-08-18

**Authors:** Giancarlo Lucchetti, Alessandra Lamas Granero Lucchetti, Daniele Corcioli Mendes Espinha, Leandro Romani de Oliveira, José Roberto Leite, Harold G Koenig

**Affiliations:** 1 São Paulo Medical Spiritist Association, Av. Juriti, 367 apto 131 – Moema, São Paulo, SP, CEP: 04520-000, Brazil; 2 Federal University of São Paulo, São Paulo, Brazil; 3 João Evangelista Hospital, São Paulo, Brazil; 4 Duke University Medical Center, Durham, USA; 5 King Abdulaziz University, Jeddah, Saudi Arabia

**Keywords:** Spirituality, Religion, Medical education, Medical schools, Medical students

## Abstract

**Background:**

According to recent surveys, 59% of British medical schools and 90% of US medical schools have courses or content on spirituality and health (S/H). There is little research, however, on the teaching of S/H in medical schools in other countries, such as those in Latin America, Asia, Australia and Africa. The present study seeks to investigate the current status of teaching on S/H in Brazilian medical schools.

**Methods:**

All medical schools in Brazil (private and public) were selected for evaluation, were contacted by email and phone, and were administered a questionnaire. The questionnaire, sent by e-mail, asked medical school directors/deans about any S/H courses that were taught, details about those courses, S/H lectures or seminars, importance of teaching this subject for medical school directors, and medical schools characteristics.

**Results:**

A total of 86 out of 180 (47.7%) medical schools responded. Results indicated that 10.4% of Brazilian Medical Schools have a dedicated S/H courses and 40.5% have courses or content on spirituality and health. Only two medical schools have S/H courses that involve hands-on training and three schools have S/H courses that teach how to conduct a spiritual history. The majority of medical directors (54%) believe that S/H is important to teach in their schools.

**Conclusion:**

Few Brazilian medical schools have courses dealing specifically with S/H and less than half provide some form of teaching on the subject. Unfortunately, there is no standard curriculum on S/H. Nevertheless, the majority of medical directors believe this issue is an important subject that should be taught.

## Background

The research on spirituality, religion and health has been increasing worldwide [1]. Studies have shown an association between religious/spiritual beliefs and both mental and physical health, treatment adherence, medical decision-making, ethical issues and values, and even survival [1-4].

There is general agreement between physicians and medical school faculty that S/H is important for patients and that health professionals should be aware and know how to deal with this aspect of whole person care [5,6].

In practice, however, few physicians address S&H issues with their patients [6]. Common barriers include lack of time, lack of knowledge, lack of training, and fear and lack of comfort in addressing this topic [5,7]. Many of these barriers are due to the failure to teach S/H in medical school.

In response to this need, the Association of American Medical Colleges (AAMC), the World Health Organization (WHO), and the Joint Commission on Accreditation of Healthcare Organizations (JCAHO) recommend that spiritual issues be addressed in clinical care and education of health professionals [8].

Additionally, a John Templeton Foundation–funded program directed by the George Washington Institute for Spirituality and Health has been established [9]. This program has a competitive award program in which medical schools proposed a curriculum in spirituality and health and the application is then judged by leading academic deans and curriculum faculty. Schools with the highest score are given a small amount of funding to develop their curricula [10].

Consequently, many medical schools have incorporated S/H into their curriculum, teaching students about the research on S/H, how to take a spiritual history, how to deal with religious conflicts, and when to refer to chaplains [9].

According to recent surveys, 59% of British medical schools [11] and 90% of US medical schools [12] have courses or content on S/H. However, little information exists on the teaching of S/H in medical schools in Latin America, Asia, Australia, or Africa [10].

Recently, Lucchetti and Granero [13] described the challenges faced in integrating spirituality in Brazilian medical schools. They reported that the resistance is largely due to the lack of Brazilian studies on S/H, trends toward secularization, and a desire to avoid religious coercion.

Another example of the challenges faced in addressing S/H in medical education were raised in an article by Peach [14] in the Medical Journal of Australia. According to the author, "religion does not play the same role in the lives of Australians as it does for US citizens (…) Spirituality has a place in Australia's medical courses, but perhaps not in practice until more data are available”.

These statements emphasize the need for more studies in different cultural and religious contexts to better understand the role of spirituality in medical students’ training.

The present study investigates the extent to which S/H is addressed in Brazilian medical schools and how it is addressed.

## Methods

A cross-sectional study was carried out from 2010 to 2011. All Brazilian medical schools (private and public) were selected based on a comprehensive list at the website http://www.escolasmedicas.com.br/ (accessed in January 2010). This site has the current status of all medical schools in Brazil and is constantly updated with contact information and names of school directors. Medical schools are also accredited by the Brazilian Ministry of Education, which has a website ( http://emec.mec.gov.br/) which lists all accredited schools.

Contact information for all schools was confirmed by checking their websites.

We then contacted all medical schools on the Brazilian Ministry of Education list. The first contact was through an email sent directly to the director responsible for medical education and to the secretary of the director. This included:

• A letter inviting participation of the institution in the study, explaining the objectives of the research, importance of participating for future curricular changes, and definitions of spirituality and religion. For the present study, spirituality was defined as “the personal quest for understanding answers to ultimate questions about life, about meaning, and about relationship with the sacred or transcendent, which may (or may not) lead to or arise from the development of religious rituals and the formation of community.” Religion was defined as “an organized system of beliefs, practices, rituals, and symbols designed to facilitate closeness to the sacred or transcendent (God, higher power, or ultimate truth/reality).” [15]

• A copy of the study’s approval by the Institutional Board Review - Federal University of São Paulo – Brazil.

• A questionnaire that asked about (1) medical school’s characteristics such as name, year started, number of students graduating per year, Brazilian federative union (state); (2) data about the person responsible for answering the survey: name, position, phone number, email; (3) types of S/H courses available: name of the course, if required or elective, if S/H was addressed in courses on other subjects, methodological aspects, duration of the courses, objectives, and whether teaching included addressing S/H issues in clinical practice; (4) S/H events: if the medical school had any S/H conferences, symposium or seminar; (5) if there were plans to start a course on S/H soon, and; (6) a question about the importance of including S/H in medical education, with the response options “not important”, “little important”, “somewhat important”, and “very important”.

**Figure 1:**
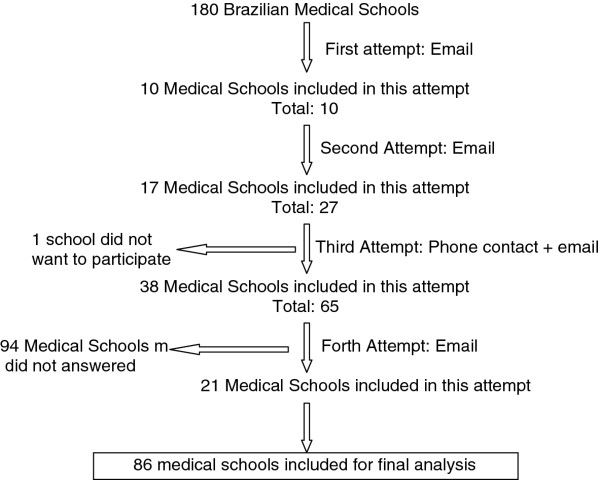
Medical school recruitment procedures.

An algorithm describing the flow of procedures in the study is presented in Figure 1. After sending the emails, all institutions had 45 days to respond to the first email. If no response was achieved, a second email was sent and another 45 days were given to respond. After the second attempt, one member of our research team phoned the medical director’s secretary, explained the objectives of the study, the importance in participating, and then sent a third email requesting confirmation of receipt (with a deadline of another 45 days to respond). We decided to extend the final deadline for another 15 days due to vacation and holidays.

Data were tabulated and analyzed through SPSS version 17 (SPSS inc.). Descriptive statistics (frequency and means) were presented.

The study was approved by the Institutional Board Review from Federal University of São Paulo – Brazil. All participants read the inform consent and gave their consent to participate in the study by completing the attached survey.

## Results

A total of 180 medical schools were registered in Brazil as of January 2010, of which 86 (47.7%) responded (Figure 1). Ten medical schools responded to the first attempt (email), 17 to the second attempt (email), 38 to the third attempt (phone contact + email), and 21 to the fourth attempt.

One institution indicated they did not want to participate because they believed this issue “was not important and the director had other things to worry about”. The other 94 institutions did not respond.

### Medical schools characteristics

All Brazilian regions were represented by participating schools; most Brazilian Federative unions (states) had about 50% of schools participate: 7 out of 17 (41.1%) schools in the North, 15 out of 38 (39.4%) in the Northeast, 20 out of 31 (64.5%) in the South, 4 out of 8 (50%) in the Center-west, 38 out of 81 (46.9%) in the Southeast, and 2 out of 4 (50%) in the Federal District.

Most institutions were private (51.7%), and the remaining were public universities (25.3% federal; 16.1% state, and 6.9% major city). Participating medical schools graduated 87.8 (SD: 44.6) students per year.

The person responsible for providing the information was: the medical director/dean in 77.9%, an associate professor in 11.6%, a medical department coordinator in 3.4%, and others in 7.1%.

#### S/H content in medical education

Of the 86 responding schools, 4 (4.6%) had a required course on S/H. In addition, 5 (5.8%) medical schools had an elective course. In summary, 9 (10.4%) Brazilian medical schools had a specific course dedicated to this subject.

In addition, 14 (16.2%) institutions reported having a S/H lecture that was given at some point in the curriculum. Another 12 (13.9%) universities reported that while they did not have a specific course or lecture, a faculty member integrated S/H in another course or lecture. Disciplines such as Ethics (in 7 medical schools), Medical Psychology (in 5 medical schools), and Community Medicine (in 5 schools) were more likely address S/H issues.

Only 4 (4.6%) medical schools indicated that they held a specific event (conference, seminar) on S/H, and only 2 (2.3%) were planning to implement a new course on S/H in their curriculum. One medical school reported online activities or online courses for their students in this area.

#### Importance of S/H in medical curriculum

When medical deans were questioned about whether their institution considered this issue important for their students, 41 (53.9%) answered “very important”, 27 (35.6%) “somewhat important”, 8 (10.5%) “of little importance,” and 0 (0%) “not important”.

#### Teaching strategies

Teaching strategies and course characteristics are presented in Table 1 for required courses and Table 2 for elective courses.

**Table 1:** Required course characteristics

**Course name**	**Main topic**	**Public or Private**	**Objectives**	**Issues**	**Learning methodology**	**Hands-on training**	**Teach spiritual history**
Religious Culture -Compulsory 36 h	Religion	Private	(a)Understand that religion is a way of seeking the truth; (b)Analyze the significance of human actions in the light of the religious phenomenon; (c) Respect different religious manifestations; (d) Emphasizing the spirit of pluralism and the question of prejudice; (e)Identify within religious traditions the processes of self-generating emancipation and solidarity	(a)The contribution of religious manifestations in the constitution of cultures and societies; (b)Recognize the characteristics of the sacred and the profane culture; (c)Approach to the relationship between health-care and salvation; (d)Approach to the death and dying - euthanasia, dysthanasia and orthothanasia;(e) Hospice care	Lectures, group discussions, debates in the classroom, library research, individual and team work, reading supplementary texts	No	No
Theology and Health -Compulsory 34 h	Religion	Private	(a) Relate theology and health in the historical, social, economic, cultural and religious practice of medicine and Christian thoughts, in a Christian and humanist perspective	(a) University and education in today's world; (b)The concept of health; (c) History of Medicine, Theology and Science; (d) Physician-patient relationship and Anthropology; (e) Jesus Christ and the Trinity; (f) The Gospel according to Luke and its relationship to health and medicine	Lectures, group discussions, supplementary internet files and movies regarding this issue	No	No
Theology and health -Compulsory 36 h	Religion	Private	(a)Think about the different interrelationships between Theology Health and Diseases; (b) Reflect on the relation between Theology and care, pain, suffering, community treatment, illness and health	(a) Concepts of Theology, Health and Illness; (b) Health as a complex process involving psycho-socio-spiritual determinants; (c) Health and social institutions: family, school, workplace and church; (d) Meaning of communication processes to human health; (e) Care in Christian practices; (e) Effect of Religiosity and spirituality in health; (f) Care and theology; (g) Caring for carers; (h) Pain and Suffering: psycho-socio-cultural and spiritual aspects; (i) Christian Therapeutic communities; (j) Jesus and the sick and excluded	Blended learning: classroom activities (inaugural and final classes) and online activities	No	No
Theology -Compulsory 2 weeks	Religion	Private	(a)Studying theology in order to incorporate their knowledge and insight for each medical act performed; (b) Assess the importance of theology and their health interaction within the medical practice; (c) Valuing the importance of the transcendental in doctor-patient relationships; (d)Valuing daily living in a multireligious world; (e) Understand the most frequent religions in Brazil; (f) Understand and respect ecumenism and religious pluralism; (g)Introduction to Theology and its importance to medical practice	(a)The multireligious world; (b) Religions in Latin America and Brazil. The foundations of the most common religions in Brazil; (c) The truths and limits of religions; (d) Ecumenism and Interreligious Dialogue; (e) Understanding the Ecumenical conception; (f) Roots of the ecumenical movement; (g) Christian Churches and ecumenical movement; (h) Organizations in defense of ecumenism in Brazil; (i) Spirituality and religious pluralism: could interfere with doctor-patient relationship?	Problem-based learning	No	No

**Table 2:** Elective course characteristics

**Course name**	**Main topic**	**Public or Private**	**Objectives**	**Issues**	**Learning methodology**	**Hands-on training**	**Teach spiritual history**
Medicine and Spirituality -Elective 60 h	Spirituality	Public	(a) Know the current state of the art of medicine and spirituality supported by scientific literature indexed; (b) know practical notions of patients’ management according to the integrative bio-psycho-socio-spiritual integrative model; (c) Personal and technical preparation in medicine and spirituality; (d) Ethical and legal principles; (e) Dealing with cultural and religious pluralism; (f) Compassion, love and medicine; (g) The limits of medicine	Not available	Theoretical classes and hands-on training. Patients visits.	Yes	Yes
Spirituality and Medicine -Elective 32 h	Spirituality	Public	(a)Realize the importance of spirituality as a factor that can impact patient's health; (b) Importance of spirituality in humanization of care	(a)Concepts of Spirituality and Religiosity; (b)Why should we study Spirituality in patient care?; (c) Reflecting on the existence of God and understanding different religions; (d) Pain and suffering in a transcendent view of human beings; (e) Spirituality in health and disease; (f) The mystery of faith; (g) Why? When? and How to include spirituality in patient care?; (h) How to address Spirituality in the hospital clinical practice; (i) The spiritual formation of the professional: a necessity or just a personal choice?; (j) Spirituality as an instrument of humanized care; (k) Research in spirituality and health: perspective of science	Lectures, group discussions, and reading supplementary texts	No	Yes
Medicine and Spirituality -Elective 20 h	Spirituality	Public	(a)Understand the human being as a spiritual being; (b) know the foundations of spirituality paradigm in medicine; (c) Be familiar with concepts of care, health and disease from the perspective of spirituality; (d) know the main lines of research in the area of spirituality and health; (e) Be familiar with the concepts in thanatology, spirituality, near-death experiences and end of life issues; (f) Encourage the doctor to form their own values concerning spirituality in health	(a) Reflection on the Myth of the Cave (Plato); (b) Studies regarding Near-Death Experiences; (c) Documentary “Life after Life” (Raymond Moody); (d) The Quantum Physics Paradigm; (e) Ethical issues and Spirituality; (f) Thanatology from a transpersonal psychology perspective	Lectures, group discussions, movies, reading supplementary texts, meditation, music therapy and shared experiences	No	No
Spirituality and health -Elective	Spirituality	Public	(a)Distinguish what spirituality, religiosity and religion are; (b) Understand, based on historical and scientific data, the role spirituality plays in the disease process, treatment, evolution, and prognosis; (c) Understand spirituality as an instrument of humanization of health care; (d) Understand the implications of spirituality in educational practice	(a)Religious life and social organization; (b) The meaning of spirituality in coping with the existential crisis brought on by illness; (c) Epidemiology of religiousness and religions; (c) Spirituality and health: a report from a visiting professor; (d) Spirituality as an instrument of humanization of health care; (e) Spirituality in educational practice; (f) Religion as a coping adaptation process into diseases; (g) Spirituality within popular education in health; (h) Implications of Spirituality in medical training	Reading supplementary texts and articles, group discussions and team work, discussion with visiting professors	No	No
Medicine, spirituality and health -Elective 30 h	Spirituality	Public	(a)Comprehensively integrate medical expertise with spiritual philosophy knowledge from different religions; (b) Expand medical teaching through religious concepts and their relation with health care	(a)Medicine, Health and Spirituality; (b) Ethical aspects in clinical practice; (c)Kirlian Photography; (d) Multidimensional anatomy and physiology; (e) New paradigms: Newtonian versus quantum physics; (f) Vibrational energies and their influence on health; (g) The concept of God; (h) Cell biology, Genetics and Spirituality; (i)Hands-on training (visiting the hospital wards); (j) Ayurvedic medicine: human consciousness and spiritual health; (k) Mind: a spiritual instrument; (l) Prayer and meditation on health; (m) Chakras: Endocrine Relations; (n) Homeopathy; (o) Humanization of medicine; (p) Hypnosis - Altered state of consciousness; (q) Conscientiology; (r) Thanatology	Lectures, seminars, group discussions, movies, questionnaire evaluations	Yes	Yes

On the one hand, required courses were more likely in private religious medical schools from Catholic and Protestant Evangelical backgrounds. These courses usually focused more on theology as it relates to culture and religion, and less on spirituality and its relationship with health. On the other hand, elective courses were more often provided in public institutions and addressed the connections between spirituality and health in a more ecumenical way.

Most courses had a 30 hour-format without students actually practicing integrating spirituality into patient care. Only two schools had courses with training in actual practice, and only three schools had courses that taught how to conduct a spiritual history.

## Discussion

Despite the growing research base on spirituality, religiousness and health [1,4], few medical schools have been addressing this issue worldwide [10].

In the present study, the first to evaluate S/H courses in a medical school from a Latin American country, we found that 10.4% of Brazilian medical schools have dedicated S/H courses and 40.5% have courses or content on spirituality and health. These results are in line with a recent Brazilian study which found that 84% psychology courses do not address spirituality in their curriculum, which underscores the challenges faced by academic institutions in including such courses in Brazil [16].

These findings are quite different compared to surveys in other countries such as United Kingdom (UK) and United States (US). In 2008, Neely et al. reported that 59% of UK medical schools had some type of course content on spirituality in their medical curriculum. Likewise, a recent survey conducted by Koenig et al. [12] showed that 90% of US medical schools have courses or content on S/H. Additionally, Fenton et al. found that 58% of US nursing schools offered content on spirituality in their curricula [17].

The results from the present study are surprising, given that Brazil is a high religious/spiritual country [18] in which 83% of the population consider religion very important in their lives, 37% attend religious services at least once a week, and 95% report an affiliation with a religious denomination. Despite this, however, there appears to be little of teaching on spirituality and health in medical curricula.

The present findings, in fact, likely represent a “best case scenario,” given that the 50% of schools not responding to our survey (despite repeated contacts) probably had even less interest in and possibly less curricular content on S/H.

What are some possible explanations for such findings? First, only a few studies on S/H were published in Brazilian medical journals prior to the year 2000. For example, in one of the most prestigious Brazilian medical databases, called Scielo (Scientific Electronic Library Online – http://www.scielo.br/), using the search word “espiritualidade”/“spirituality” we found 0 articles in 1999, 7 articles in 2005, and 18 articles in 2010. Second, there is resistance to the introduction of these courses due to the view that medicine should be kept secular and therefore avoid addressing religious/spiritual issues because this may be experienced as coercive by some patients [13]. Third, few Brazilian medical conferences addressed S/H before the year 2000, university research departments seldom investigated S/H, and only one post-graduate program on S/H exists in the entire country.

We also found a notable difference between private and public medical schools. Private schools were more likely to have required courses that focused on theological issues related to culture and religion (Table 1), whereas public medical schools were more likely to have elective courses that focused on connections between spirituality and health from a broader perspective (Table 2). According to the Association of American Medical Colleges, medical curricula should provide students with an understanding of the role that spirituality plays in the care of patients in different clinical situations and the effect their own spirituality has on their ability to provide compassionate care that involves the spiritual aspects of patients’ lives [8].

These objectives are more attuned to the courses provided by public medical schools than private ones in Brazil. Some knowledge regarding different religions, however, is important for ethical and legal reasons, as well for addressing the religious needs of patients which often surface when patients become ill (particularly in a country as religious as Brazil). A spiritual history, then, should inquire about the specific religious beliefs, practices, and needs of patients, as well as about the broader spiritual aspects of patients lives and needs that arise from them.

A key objective of AAMC is that all students should be able to take a spiritual history as part of the medical history [8]. Unfortunately, in our study, only two schools included hands-on practice on how to integrate spirituality into patient care, and only three taught how to conduct a spiritual history. These findings are in line with S/H courses in UK [11], where only a few medical schools teach students how to take a spiritual history.

The small proportion of Brazilian medical schools that teach spiritual history taking and provide hands-on training should be emphasized, since doing so is important to achieve a better understanding of how spirituality influences the patient’s health [6,10,19]. There are several instruments which can guide the physician in taking a spiritual history [20] and which can help overcome the barriers to addressing spiritual issues (lack of knowledge, lack of training, fear of imposing religion) [5]. Nevertheless, the lack of hands-on training may leave the medical student without practical skills when encountering real patients.

Another concern is the lack of uniformity in S/H curricular content. Some courses are dedicated exclusively to religious issues (“Jesus Christ and the Trinity”, “the contribution of religious manifestations in the construction of cultures and society,” etc.); others focus on complementary and alternative medicine (CAM) practices such as Reike, ayurvedic medicine and hypnosis; and still others emphasize the interface between quantum physics and connections to health (through Kirlian photography, bioelectrography, near-death experiences, etc.). All of these topics are considered “spiritual.” This situation reflects a lack of specific training for faculty, a lack of consensus between faculty on what should be included in the curriculum and lack of a national policy with regard to S/H in medical education.

It is also possible, however, that the medical deans we surveyed may not have always been aware of courses that included S/H content, since they may not have been involved in those courses and instead relied on what their often large and diverse faculty shared with them. In a recent consensus conference on palliative care, spirituality was considered a fundamental component of palliative care [21]. Likewise, CAM courses often include aspects of S/H in their content, which respondents to our survey may not have been aware of [22].

Interestingly, the majority of medical directors (54%) believed that this issue is important for their schools and none reported that it was not important. These results are even more positive than those from a study of US medical school deans [12], of whom less than 40% felt that introducing S/H contents into the curriculum was important and only 10% indicated their faculty valued S/H content as “very valuable.” We should note that there is a clear difference between medical school deans’ opinions and their medical school's actual practices. While medical school deans in Brazil believe this issue is important, they often do not integrate it into the curriculum. We understand that a major barrier to doing so involves the intense competition for time in the curriculum, the lack of research on S/H in Brazil, and lack of qualified faculty to teach such courses.

Despite the limited space in the curriculum, addressing spiritual issues related to clinical care should be given more attention.

There is a general agreement that spiritual/religious beliefs can impact physical and mental health as well as affect ethical, legal and medical decisions [3,15]. There is also agreement that physicians should not prescribe religious activities that patients are not already doing [23]. There is no consensus, however, on whether physicians should pray with patients [24], what kind of issues should be taught in S/H course, and how these courses should be evaluated [10].

Our study has a number of limitations. First, despite substantial efforts to achieve a high response rate (sequential emails, phone contacts, expanded deadlines), we received responses from only about half of Brazilian medical schools. As noted earlier, this may represent a lack of interest by some schools in the topic. Nevertheless, our response rate was similar to Neely et al. [11] who evaluated UK medical schools. Second, some medical directors/representatives may not have been aware of S/H initiatives conducted by their faculty and therefore chose not to respond. Third, the questionnaire did not include a number of factors that could affect the inclusion of S/H in the medical curriculum, such as curricular time, funding, training support, questions about curricular competencies, methods to evaluate these competencies, and type of teamwork available within the school. In order to maximize the response rate, these questions could not be included.

## Conclusion

In conclusion, few Brazilian medical schools have courses dealing specifically with S/H and approximately 40% currently provide some form of teaching on the topic. Unfortunately, most teaching on S/H is not standardized and few schools include the opportunity for students to actually practice what they learn. Nevertheless, it is encouraging that more than 50% of medical directors feel that S/H is an important aspect of patient care that students should be aware of.

## Competing interests

The authors declare that they have no competing interests.

## Authors’ contributions

GL: substantial contributions to conception and design, acquisition of data, analysis and interpretation of data; drafting the article and final approval of the version to be published. ALGL: substantial contributions to conception and design, interpretation of data; revising the article critically for important intellectual content; and final approval of the version to be published. DCME: acquisition of data, revising the article critically for important intellectual content; and final approval of the version to be published. LRO: substantial contributions to conception and design; revising the article critically for important intellectual content; and final approval of the version to be published. JRL: substantial contributions to conception and design, revising the article critically for important intellectual content; and final approval of the version to be published. HGK: Interpretation of data; revising the article critically for important intellectual content; and final approval of the version to be published. All authors read and approved the final manuscript.

## Pre-publication history

The pre-publication history for this paper can be accessed here:

http://www.biomedcentral.com/1472-6920/12/78/prepub
